# Role of Vitamin A in Mammary Gland Development and Lactation

**DOI:** 10.3390/nu12010080

**Published:** 2019-12-27

**Authors:** M. Teresa Cabezuelo, Rosa Zaragozá, Teresa Barber, Juan R. Viña

**Affiliations:** 1 Department of Physiology, Universitat de València, Avda. Blasco Ibañez, 15, 46010 Valencia, Spain; tecabar@alumni.uv.es; 2 University Hospital Doctor Peset, Gaspar Aguilar, 90, 46017 Valencia, Spain; 3 Department of Human Anatomy and Embryology-INCLIVA Biomedical Research Institute, Universitat de València, 46010 Valencia, Spain; 4 Department of Biochemistry and Molecular Biology-INCLIVA Biomedical Research Institute, Universitat de València, 46010 Valencia, Spain; teresa.barber@uv.es (T.B.); juan.r.vina@uv.es (J.R.V.)

**Keywords:** vitamin A, retinoic acid, vitamin A deficiency, lactating mammary gland, weaning, involution

## Abstract

Vitamin A (all-*trans*-retinol), its active derivatives retinal and retinoic acid, and their synthetic analogues constitute the group of retinoids. It is obtained from diet either as preformed vitamin A or as carotenoids. Retinal plays a biological role in vision, but most of the effects of vitamin A are exerted by retinoic acid, which binds to nuclear receptors and regulates gene transcription. Vitamin A deficiency is an important nutritional problem, particularly in the developing world. Retinol and carotenoids from diet during pregnancy and lactation influence their concentration in breast milk, which is important in the long term, not only for the offspring, but also for maternal health. In this study, we review the role of vitamin A in mammary gland metabolism, where retinoid signaling is required not only for morphogenesis and development of the gland and for adequate milk production, but also during the weaning process, when epithelial cell death is coupled with tissue remodeling.

## 1. Introduction

Vitamin A, an isoprenic-derived micronutrient, strictly refers to the alcoholic form all-*trans*-retinol, although in a broad sense includes both its active derivatives as well as other synthetic analogues that exhibit its biological actions. All of these molecules constitute the group of retinoids [1]. Their important role has been demonstrated through many scientific approaches and clinical observations. Retinoids are required for embryonic development and exert important effects on postnatal physiological events, including cell differentiation and proliferation, immunity, ocular function, and reproduction, and are also important antioxidants [2,3,4,5,6,7,8,9]; however, it must also be considered that treatment with retinoic acid (RA) can increase oxidative stress [10].

Retinol is a liposoluble micronutrient essential in our diet. It is available in foods either as preformed vitamin A; as retinyl ester (RE), abundant in some animal-derived sources such as liver, eggs, dairy products, and fatty fish; or as provitamin A carotenoids, mainly β-carotene, abundant in dark colored fruits and vegetables such as green leaves, carrots, ripe mangos, and other orange–yellow vegetables. This heterogeneous group of carotenoids presents other healthy effects as well, including antioxidant, anti-inflammatory, and immunomodulatory properties [11]. Dietary vitamin A is absorbed in the small intestine in the form of retinol and transported in blood attached to retinol-binding protein (RBP). Inside the cells, retinol is oxidized into its main biologically active derivatives, first retinaldehyde (retinal), which plays a role in vision, and then retinoic acid (RA), which regulates the expression of multiple target genes. Most functions of vitamin A are mediated through the RA activation of two types of transcription factors, retinoic acid receptors (RARs) and retinoid X receptors (RXRs), each with three subtypes (α, β, and γ) and each of those with different isoforms [12]. All-*trans*-RA (atRA) is a high-affinity ligand for RARs, while 9-*cis*-RA binds to RARs and RXRs. Upon binding to RA, RAR–RXR heterodimers bind to the RA response element (RARE) in the promoter regions of target genes and stimulate their transcription. Moreover, RA also has extranuclear, nontranscriptional effects, which directly influence the expression of several genes through phosphorylation processes. Different transcriptional effects of retinol and retinal have also been extensively described [12,13,14,15].

According to the World Health Organization (WHO), vitamin A deficiency (VAD) is a public health problem in 50% of countries; it is the most common nutritional disorder in the world, along with protein malnutrition. The leading cause of VAD in humans is deficient dietary intake, which occurs particularly in developing countries and may be exacerbated by high rates of infection, especially diarrhea and measles. In low- and middle-income countries, where intake of animal-based foods is low, dietary provitamin A carotenoids are the main source of vitamin A, and higher amounts are needed to meet the requirements of this vitamin. VAD might be aggravated during the stages of life in which nutritional demands are greater, such as pregnancy, lactation, and early childhood [16]. Dietary deficiency can start early in life, with the colostrum being discarded or with inadequate breastfeeding, which means that babies are deprived of their first important source of vitamin A. Mothers from poorer populations, where intake of vitamin A and carotenoids is marginal, have a much lower content of these micronutrients in their milk compared to mothers from developed countries. It is important to consider that nutrients provided through maternal milk derive not only from the diet during pregnancy and lactation, but also from maternal reserves. In this sense, the potential long-term effects of nutrient depletion during any of these periods are important not only to fetal and child development, but also to maternal health [11,16,17,18,19].

This review focuses on the role of vitamin A in the mammary gland along each developmental stage. First, it covers the importance of retinoids in the morphogenesis and development of the gland. Next, we note that during lactation, it is essential to meet the requirements to fulfill an adequate milk production. Finally, we also consider the role of this micronutrient in the weaning process, when extensive tissue remodeling occurs and epithelial tissue regresses to return the gland to a structure similar to the pre-pregnant state, thus the tissue is prepared for the next pregnancy and lactation. We also cover the impact of vitamin A deficiency on both the mother and her offspring’s health during the period of pregnancy and lactation, when the most important stages of development in human life occur.

## 2. Vitamin A Dietary Sources, Availability, and Requirements

Dietary vitamin A is obtained in mainly two forms, as preformed vitamin A or as provitamin A carotenoids. Preformed vitamin A is mainly found as long-chain fatty acid esters of retinol, particularly retinyl esters (REs), which are easily hydrolyzed endogenously to form retinol. Preformed vitamin A is found in animal sources such as eggs, fish oils, organ meats, and dairy products and their derivatives, although liver is considered the source with the greatest quantitative importance. On the other hand, the provitamin A carotenoids, polyisoprenoid pigments of vegetable origin, are partially converted to vitamin A in the intestinal mucosa. To date, more than 750 carotenoids have been identified, although only about 40 of them are consumed in substantial amounts in the human diet, the most abundant being β-carotene, lycopene, lutein, β-cryptoxanthin, α-carotene, and zeaxanthin [20]. Not all carotenoids exhibit provitamin A properties; only 10% of natural carotenoids can be metabolically cleaved to produce at least one molecule of retinol. The main structural requirement of a carotenoid for its provitamin A activity is the presence of at least one ring of unsubstituted β-ionone with a polyene chain of 11 or more carbon atoms [21,22]. As previously mentioned, only a few of these precursors with provitamin A activity are found in significant amounts in the human diet, mainly β-carotene, with minor contributions from α-carotene, β-cryptoxanthin, β-canthaxanthin, and β-echinenone. The dietary sources of these carotenoids are colored fruits (orange, apricot, mango, etc.) and some vegetables and their formulations (carrot, tomato, sweet potato, pumpkin, broccoli, cabbage, spinach, red palm oil, etc.) [23,24,25]. Other carotenoids commonly found in foods, such as lycopene, lutein, and zeaxanthin, do not meet these structural requirements and are not precursors of vitamin A.

Dietary sources of vitamin A differ among countries, due to not only economic but also cultural factors. In Western countries, approximately 75% of vitamin A is ingested as preformed REs and comes mainly from dairy products, or fortified products such as breakfast cereals, butter or margarine, and infant formula. On the other hand, in developing countries, 70–90% of vitamin A is consumed as provitamin A carotenoids. Therefore, in populations in low-income countries where vitamin A is mainly consumed in the form of carotenoids, the risk of VAD increases [16,26]. Moreover, in low-income countries with a rice-based diet, VAD is aggravated, since rice lacks this vitamin. Consequently, in some developing countries, vitamin A is added to certain products, such as sugar, thus improving the vitamin A status in the global population. Several successful efforts have been made to improve the provitamin A content in major crops such as wheat, rice, and potato, which are poor in β-carotene (e.g., “golden” rice or “golden” potato tubers that overexpress three bacterial genes for β-carotene synthesis) [27,28,29,30]. In spite of these modifications, the prevalence of subclinical deficiency is increasing [25]. However, it also has to be taken into account that VAD is not exclusive to developing countries, as it can also occur in Western societies depending on different factors such as a highly restrictive diet [16,31,32,33,34,35,36].

The bioavailability of vitamin A in food is defined as the ingested fraction available for use and storage, and depends on the capacity of the digestive process to release it from its original matrix in the food and on the fraction, which, once absorbed, is converted into retinol. Vitamin A bioavailability is higher in foods of animal origin (retinyl esters, preformed vitamin A) compared to vegetable origin (provitamin A carotenoids), although some of the latter group are considered good sources of vitamin A. Based on the efficiency of absorption and conversion into vitamin A, 1 µg of all-*trans*-retinol is equivalent to 12 µg of β-carotene and 24 µg of α-carotene or β-cryptoxanthin. However, the efficiency of β-carotene to be converted into retinoids is probably worse than previously thought, particularly in developing countries where vitamin A status and several other factors can reduce its conversion efficiency [31,36]. Accordingly, the use of a 21:1 ratio for mixed diets (12:1 for fruits and 26:1 for vegetables) has been suggested [25,36]. Thus, compared with preformed vitamin A, provitamin A carotenoids are relatively poor but important sources of vitamin A for the global population, as a higher amount is needed to meet vitamin A needs. On the other hand, it is important to consider for all groups of populations that several factors can influence the bioavailability of vitamin A from the diet, including the presence and severity of infections and parasites, intestinal or liver disease, iron and zinc status, xenobiotics, levels of dietary fat, protein malnutrition, alcohol intake, dietary source (preformed or provitamin A carotenoids), and food processing [11,16,31,35,37,38,39,40].

The requirements for vitamin A are expressed in retinol activity equivalent (RAE): 1 µg of RAE is equal to 1 µg of all-*trans*-retinol. In terms of international units (IUs), 1 IU is equal to 0.3 µg of all-*trans*-retinol or 0.3 µg of RAE. The recommended dietary allowances (RDAs) for children, men, and women are 300–600, 900, and 700 mg of RAE/day, respectively. However, the demand for micronutrients increases to 750 mg of RAE/day during pregnancy and 1300 mg of RAE/day during lactation. The gestational period is nutritionally relevant for maternal, fetal, and infant health. During the breastfeeding period, lactating mothers are susceptible to vitamin deficiency as the neonate feeds on her stores through the milk. Vitamin A is one of the most critical micronutrients in this period, affecting lung function and maturation, and thus susceptibility to infection. Inadequate maternal intake of vitamin A translates to an inadequate supply to the fetus during pregnancy and to the neonate during lactation through breast milk [37,41,42,43,44]. It is noteworthy that this deficiency during embryogenesis and the early months of life cannot be compensated in the postnatal period. Furthermore, in humans, an insufficient supply of vitamin A to the fetus is associated with organ malformations, preterm birth, and low neonatal stores, which increase the risk of infectious diseases and could exert a negative effect on health later in life [36,45,46,47]. Pregnant and breastfeeding women are considered to be at risk due to their higher demand for micronutrients, and should be advised to consume enriched products with β-carotene or, even better, vitamin A plus β-carotene, in order to avoid nutritional deficits [16,19,25,31,32,33,34,48].

In contrast to what was established in the case of vitamin A, there is no dietary intake recommendation for carotenoids for any population group. The average intake of β-carotene in Western societies is 3.9 mg/day and the total intake of provitamin A carotenoids is approximately 5.2 mg/day, higher in woman than men. The maximum daily amount of β-carotene that the adult intestine can cleave is approximately 2.5 mg, and the minimum amount of fat required for optimal absorption of carotenoids is about 3–5 g per meal [31,34,41,45].

## 3. Intertissue Flux and Metabolic Transformations of Vitamin A

Vitamin A, mainly found in the diet as REs, is hydrolyzed in the lumen of the small intestine into retinol molecules. This free retinol enters the enterocytes by a carrier-mediated process and by passive diffusion. On the other hand, vitamin A precursors, carotenoids, cross the epithelial cells by membrane-bound transporters and are metabolized within the cells to retinal, which can then be converted to retinol. Absorption of vitamin A appears to be fairly high and more significant than that of carotenoids. However, due to their hydrophobic nature, the absorption of both preformed and vitamin A precursors depends on micellar solubilization and thus on the fat content of the diet [25].

**Figure 1:**
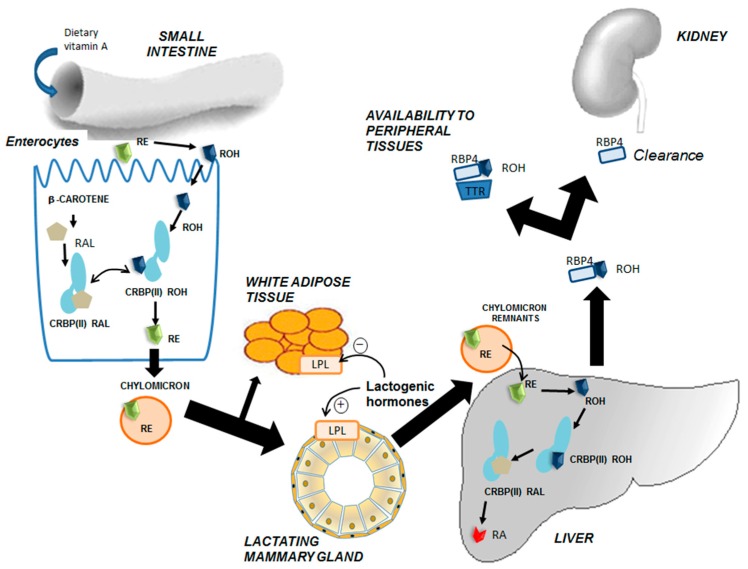
Intertissue flux of vitamin A during lactation. Dietary intake of vitamin A occurs mainly in the form of precursor β-carotene from vegetables and fruits, or retinyl esters (REs) from sources of animal origin. Both molecules are absorbed by enterocytes in the small intestine, although REs must be hydrolyzed into retinol (ROH) within the intestinal lumen by means of hydrolases. Within enterocytes, ROH and β-carotene are metabolized into REs, which in turn are secreted into the lymph in chylomicrons. Circulating chylomicrons, before reaching the liver, are hydrolyzed in tissues with lipoprotein lipase (LPL) activity and REs released into these extrahepatic tissues. During lactation, increased levels of lactogenic hormones regulate LPL activity, inducing it in the mammary tissue, whereas it is reduced in white adipose tissue. The effect of these hormones redirects REs into the mammary epithelial cells during lactation. Finally, chylomicron remnants are taken up by hepatocytes, where REs are hydrolyzed into ROH, stored, or oxidized to retinoic acid (RA). When needed, ROH is released from hepatic cells into plasma bound to retinol-binding protein (RBP4). Once in plasma, it associates with the stabilizing protein transthyretin (TTR), which decreases the renal clearance of vitamin A [49,50,51,52,53,54,55].

After absorption, retinol is re-esterified to REs, incorporated into chylomicrons together with a small fraction of carotenoids, and transported for a short time in plasma. Finally, the action of lipoprotein lipase (LPL) in different tissues affects chylomicron remnants that contain REs. Those chylomicron remnants are directed mainly to the liver in a receptor-mediated process (low-density lipoprotein (LDL) receptor, or via LDL-related protein, LRP). REs are then hydrolyzed back to retinol in the liver, which is secreted into plasma associated with retinol binding protein (RBP4), oxidized to RA, or transferred to stellate cells for storage mainly as esters of palmitic acid. Thus, the liver serves as the main pool for vitamin A (Figure 1) [23,40,49,50,51,52]. When needed, the vitamin A reserve is mobilized and secreted as the all-*trans*-retinol–RBP (holo-RBP4) complex to circulating blood. This complex associates with transthyretin (1:1) in plasma, reducing the glomerular filtration of retinol in the kidneys.

Retinol is delivered from plasma to extrahepatic cells by free diffusion or a receptor-mediated process (stimulated by RA 6, STRA6) [56]. Once inside the cells, retinol is reversibly oxidized to retinal by enzymes of the retinol dehydrogenase complex and short-chain dehydrogenase/reductase (SDR/RDH) families. Retinal is irreversibly oxidized to RA by cytosolic aldehyde dehydrogenase 1 (ALDH1) isoenzymes. RA regulates gene transcription by binding to nuclear retinoid receptors (RARs and RXRs), which act as transcription factors and can be further oxidized for excretion, catalyzed by mono-oxygenases of the CYP family. There are cellular binding proteins for retinol and retinal (CRBP) and RA (CRABP), which enable their metabolism and action [25,40,49,56].

## 4. Role of Vitamin A during Development and Regression of the Mammary Gland

Vitamin A has several pleiotropic effects in cell differentiation, embryogenesis, apoptosis, etc. Retinoic acid and its precursor retinol are known to be involved in the maintenance, differentiation, and function of many epithelial tissues, among which the mammary gland is included.

### 4.1. An Overview of Mammary Gland Development

The mammary gland structure consists of a compound tubulo-alveolar gland embedded within irregular connective tissue. This complex organ undergoes a series of changes from conception to senescence, which are regulated by hormonal activity, mainly prolactin, estrogen, and progesterone. The peculiarity of this highly dynamic glandular tissue lies in the fact that it reaches full development only after birth. During the lifetime of the female, the mammary gland will suffer profound changes in structure and function to adapt to the physiological processes of pregnancy, lactation, and involution. Moreover, with each menstrual cycle, the mammary gland undergoes a round of expansion, proliferation, and regression under the influence of sexual hormones. Along these different stages, the subpopulations of cells that form mammary tissue will proliferate, differentiate, or even die, giving rise to significant remodeling of the glandular architecture [57].

At birth, a rudimentary embryonic ductal tree is present and will remain quiescent until puberty. At this point, under the control of sexual hormones, ductal epithelium develops and grows, invading the mammary fat pad in a process known as branching morphogenesis. Finally, the gland reaches a bilayered ductal tree structure, where luminal epithelial cells form the alveoli, with their apical side toward the lumen and basal side surrounded by myoepithelial contractile cells. Throughout adulthood, the epithelium proliferates and regresses during each estrous/menstrual cycle [58]. However, it is during pregnancy and lactation when the most dramatic changes occur: Proliferation of the alveolar epithelium concomitant with differentiation into secretory alveoli capable of producing milk. During lactation, oxytocin released by suckling stimuli will trigger the contraction of myoepithelial basal cells, and thus milk ejection toward the nipple. Then, after weaning, secretory epithelial cells are no longer needed and there is regression of the epithelial compartment, together with re-differentiation of adipocytes. This process, termed involution, includes programmed cell death and remodeling of the mammary gland and extracellular matrix (ECM), mainly driven by metalloproteases, returning the gland to the pre-pregnant state. Finally, during the postmenopausal period, the breast undergoes a second process of involution, characterized by atrophy of the parenchymal structures [59].

The mammary gland is a unique experimental model to study all of the physiological changes that occur along the different developmental stages: Proliferation, branching morphogenesis, differentiation, cell death, regression, and adipocyte differentiation. In this sense, several murine models have been extensively used to decipher the underlying mechanisms controlling all of these processes [60,61,62,63,64,65,66,67,68,69,70].

### 4.2. Retinoids and Mammary Gland Development

RA is an important regulator of cell differentiation and plays a major role in embryonic development and tissue remodeling [71]. The biological action of RA is mediated by three nuclear receptors, RARα, β, and γ, which are ubiquitously expressed in the breast. Moreover, several in vivo and in vitro studies have shown that RA is required for mammary gland morphogenesis. RA signaling is involved in initiating mammary gland formation, and precise levels of RA are crucial for normal mammary epithelium development during embryogenesis. Indeed, the expression of several genes involved in RA signaling, such as retinaldehyde dehydrogenase 2 (*Raldh2*) or RARβ, has been detected in mouse embryos at day 10.5. In cultured mouse flanks, the expression of genes involved in mammary gland development could be modulated by retinoic acid signaling. It is interesting to point out that during embryonic development, the mammary epithelium was inhibited by both increased and decreased levels of RA, reinforcing the importance of a narrow range for this molecule to exert its physiological function [60].

Nevertheless, the role of retinoids within the mammary gland is not restricted to the early embryonic stages when rudimentary mammary gland is formed. On the contrary, retinoid signaling regulates almost all developmental stages of the mammary tissue. Several studies have shown that retinoic acid signaling is required for development of the mammary gland after birth. The effects of retinoids are mediated through RARs and RXRs, which are differently expressed in distinct phases of mammary gland development [61]. Indeed, RARα controls mammary development before and during puberty, and loss of this nuclear receptor has a major effect on expansion of the neonatal ductal tree and branching morphogenesis in pubertal mice [61,62]. Similarly, in adult human breast tissue, RARα is also ubiquitously expressed in the nuclei of mammary epithelial cells, and the effects of RA seem to be controlled by the expression of *Raldh1*, which converts retinol to RA. In fact, in primary cultures from human mammary epithelial cells, when this gene was knocked down, mammosphere formation was significantly reduced. This effect could be rescued by treating the cells with RA, suggesting that the retinoic acid pathway is essential for mammosphere formation and thus for branching morphogenesis [63].

Analogous results were obtained in murine models of pregnancy and lactation. Abrogation of the RA signaling pathway in transgenic mouse models led to mammary gland hyperplasia [61,64] and excessive branching morphogenesis, and these mice displayed lactational deficiency. These effects of VAD deficiency could be reverted by RA supplementation, confirming that vitamin A is essential for mammary gland development at both nulliparous and pregnancy/lactation stages [61]. Further reinforcing these results, it was shown that RA induced lumen formation in vitro in a two-dimensional (2D) model where mammary epithelial cells were grown in collagen gels [65], and treatment of MCF-10A cells with RA induced re-differentiation of these cells [66]. The role of vitamin A in lumen morphogenesis is by mediation through the RARα signaling pathway, extending beyond the intracellular effects, since extracellular matrix remodeling is required for lumen formation. Moreover, RA deficiency in mice shows stromal hypercellularity and increased collagen, altering homeostasis in the ECM [64]. All of these findings therefore indicate that retinoids play an important role in the development and maintenance of glandular epithelial structures.

### 4.3. Retinoid Signaling During Mammary Gland Involution

**Figure 2:**
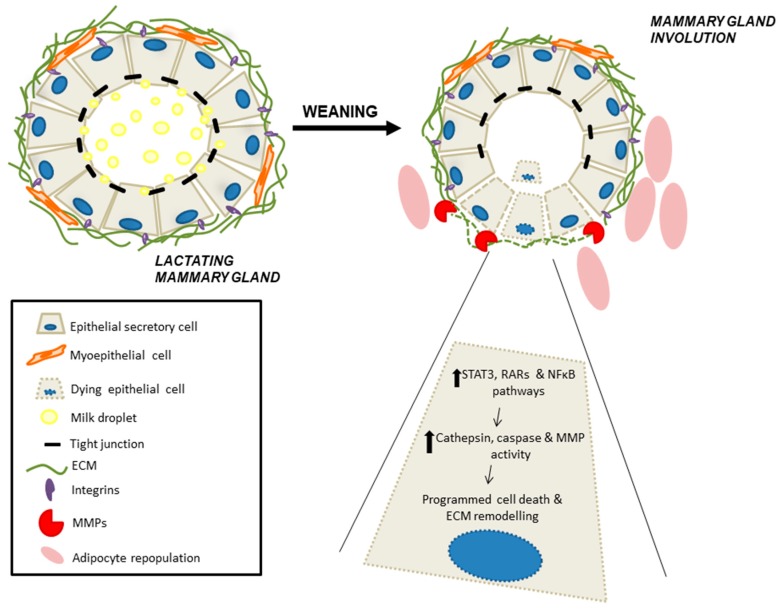
Schematic model of mammary gland involution. Lactation is characterized by an alveolar architecture lined by secretory mammary epithelial cells and contractile myoepithelial cells underneath. After weaning, milk stasis within the lumen and other local factors initiate involution of the mammary gland. At this point, cell shedding and cell death (cells with dotted lines) begins by 12 h after milk stasis, but lactation is still reversible until 48 h of weaning. Beyond this time point, epithelial tight junctions are disrupted, followed by extracellular matrix (ECM) remodeling and adipocyte re-differentiation [68,70,72,73]. MMP, metalloprotease; RAR, retinoic acid receptor; STAT3, signal transducer and activator of transcription 3; NF-kB, nuclear factor kappa B.

At the end of lactation, following weaning of the offspring, the mammary gland undergoes massive cell death and tissue remodeling with regression of the epithelial structures in order to return to a virgin-like state. Involution is characterized by three major events: Programmed cell death of epithelial cells, degradation of the basement membrane and ECM remodeling, and, finally, adipocyte repopulation with a concomitant loss of the lobular–alveolar structure (Figure 2) [68,69,70]. Several signaling pathways are known to modulate mammary gland involution. Milk stasis within the gland activates programmed cell death of epithelial secretory cells that are no longer needed; this process is ruled by STAT3 and NF-κB transcription factors, which are rapidly activated upon cessation of suckling stimuli [68,72,73].

One could hypothesize that since milk production has ceased during this stage and blood flow to the gland also decreases, vitamin A recruitment to the gland would also be reduced, thus inhibiting the whole signaling pathway. Surprisingly, vitamin A plays an important role during weaning, not as a micronutrient to be secreted within milk, but as a signaling molecule instead. The RA signaling pathway is fully activated during mammary gland involution. Cytoplasmic transporter CRABP II and CRBP-I carrier proteins, together with several isoforms of RAR and RXRα, are significantly increased during involution. Besides, as previously described, RARα exerts its effect by binding to gene promoters such as the metalloprotease 9 (MMP-9) promoter, which induces MMP-9 mRNA and protein levels and, finally, proteinase activity [67]. The role of retinoids in matrix remodeling is reinforced by the fact that administration of an acute dose of retinol palmitate to control lactating rats also induced MMP-9 expression. This emphasizes the importance of retinoids in vivo to regulate mammary gland involution [67,68].

## 5. Vitamin A and Carotenoid Uptake by the Mammary Gland during Lactation

Vitamin A is transferred to the offspring by limited placental transfer during gestation, and mostly through maternal milk during the neonatal period. Because of the limited transplacental transfer, mammal newborns have low stores of vitamin A, depending significantly on the lactation period to acquire enough reserves and maintain adequate growth and development [37,44,45,74,75,76,77,78,79].

**Figure 3:**
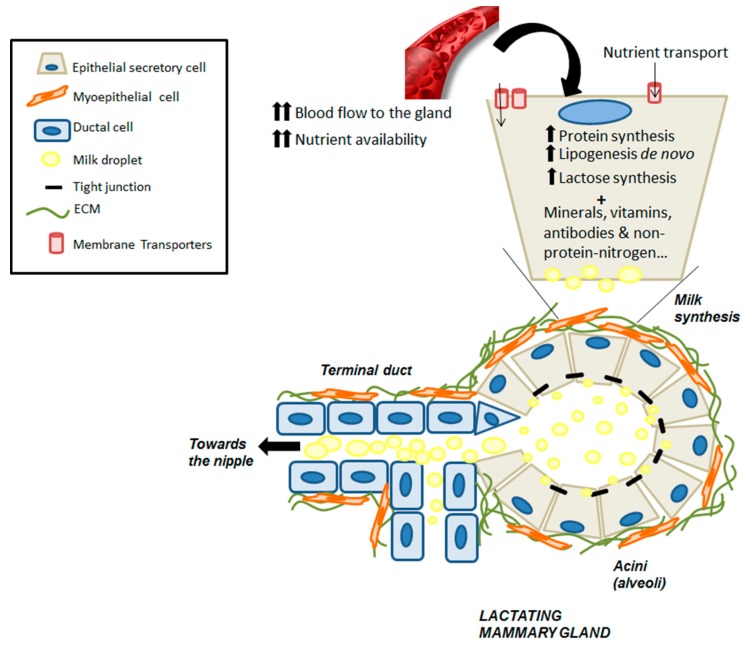
Metabolic events that lead to milk synthesis in alveolar cells from lactating mammary gland. During lactation, increased blood flow and intertissue redistribution of nutrients toward the mammary gland, together with increased membrane transporters, ensure bioavailability of precursors for milk synthesis within the epithelial cells. Both lipid and carbohydrate anabolism are increased, as is protein synthesis. Within the apex of the alveoli, milk-lipid droplets are formed and released toward the lumen with other milk components such as vitamins, minerals, and salts [81,82,83,84,85].

Lactation is characterized by widespread changes in the metabolism of different tissues to ensure a sufficient supply of nutrients to the mammary gland for milk production [80]. Among these changes, LPL in adipose tissue is inhibited; meanwhile, this enzyme is activated in the mammary tissue, therefore triglycerides are redirected to the latter [53,54,55]. Regarding amino acid and glutathione metabolism, there is also redistribution from the liver to the mammary gland, together with a nitrogen-sparing mechanism [81,82,83,84,85]. In addition, several physiological changes are required to ensure proper milk production, such as hyperphagia, liver and mammary gland hypertrophy, increased cardiac output, and increased blood flow to the gland. In this sense, substrate uptake by the lactating mammary gland is determined by several factors: (1) Increased availability of the substrates, which is met by hyperphagia and increased blood flow and substantial redistribution from different tissues to the mammary gland [81,82]; (2) transport mechanisms with increased expression of several carriers [85]; and (3) increased metabolism of nutrients within the gland [83,84]. All of these factors are under tight regulation by local and general effectors and contribute to milk production (Figure 3).

Vitamin A sources for the mammary gland comprise REs and carotenoids packed in the chylomicrons synthesized from dietary intake, and serum retinol (RBP4–retinol), which does not vary over a wide range of vitamin A ingestion and will be esterified within the mammary gland. During lactation, hyperphagia allows for higher vitamin A and carotenoid intake, which is associated with increased redistribution to the mammary tissue and thus to the secreted milk. REs associated with chylomicrons from the diet travel through the lymph and plasma, reaching extrahepatic tissues before getting to the liver. During breastfeeding, a great proportion of this vitamin A from the diet is redirected, preferably to lactating mammary tissue, as the activity of LPL in the mammary gland is a determinant for this highest uptake by the gland [53,54,55]. Indeed, the higher activity of LPL in thr mammary gland and the depressed activity in other tissues, such as adipose tissue, triggers the transfer of fatty acids, partial triglycerides, some unesterified cholesterol, and fat-soluble vitamins to the parenchymal cells of the gland during lactation (Figure 1). Once inside the mammary secretory epithelial cells, retinol is found to be bound to CRBPs, which will facilitate metabolism or excretion of this compound. In fact, it has been reported that CRBP-I and CRBP-III are both expressed in the mammary gland at different developmental stages. CRBP-III-null mice showed reduced RE levels in milk, suggesting that this isoform plays a role in retinol metabolism within lactating mammary tissue [86]. Human-milk vitamin A is present almost exclusively as REs, mainly retinyl palmitate, in the lipid fraction of the milk.

Hormonal changes are responsible for this nutrient redistribution and higher uptake within the mammary tissue. Preceding parturition, a number of hormones (insulin, steroids, and prolactin) play a role in preparing the gland for lactation [76]. Several studies using animal models have elucidated the role of these hormones in the intertissue flux of nutrients, together with the metabolic adaptations that occur during lactation [53,54,55,80,83,84,85]. During breastfeeding, the regulation of metabolism in lactating rats, and in particular milk production, appears to depend mainly on two hormones, prolactin and insulin. The insulin/glucagon ratio in lactation is decreased, but the number of insulin receptors, especially those with high affinity for the hormone, are increased in lactating mammary gland, contrary to what happens in adipocytes and hepatocytes, where the number of insulin receptors does not change. In this context, it is important to highlight that mammary tissue lacks glucagon receptors, and consequently, glucagon cannot affect mammary gland metabolism. On the other hand, prolactin levels in serum, which are already increased at the end of gestation, enhance LPL activity within the mammary gland and negatively regulate LPL activity within adipocytes, allowing for increased metabolism of RE-chylomicrons in mammary tissue. Overall, these studies highlight that high plasma prolactin levels are mainly responsible for the cooperative changes in the metabolism of different tissues during lactation that preferably redirect the circulating substrates to the mammary gland for milk synthesis; insulin is also likely to play a role in this intertissue flux of nutrients [25,53,54,55,76,80,83,85].

## 6. Vitamin A Concentration in Milk

Maternal milk is considered to be the optimal source of nutrition for infants and contains all essential vitamins. Accordingly, international organizations (e.g., WHO, UNICEF, American Academy of Pediatrics (AAP), European Society for Paediatric Gastroenterology Hepatology and Nutrition (ESPGHAN)) recommend exclusive breastfeeding during the first 6 months of life [17,41,44]. It is species-specific and a complete food adapted to the requirements for the survival and healthy development of offspring, by providing macronutrients (fats, carbohydrates, proteins, and free amino acids), micronutrients (vitamins and minerals), protective factors, and other important components for growth, such as cytokines, oligosaccharides, growth factors, and hormones [17,42,75,76,77]. A recent meta-analysis concluded that breast milk is not only a perfect nutritional supply for the infant, but is also probably the most specific personalized medication, at a time in life when genetic expression is being achieved. Breastfeeding provides protection against childhood infections and malocclusion, and is probably related to reductions in instances of overweight and diabetes, and globally could prevent 823,000 deaths of children younger than 5 years annually [87]. However, even in low- and middle-income countries, where breastfeeding duration is longer than in high-income countries, only 37% of children under 6 months of age are exclusively breastfed [87].

The milk produced in the first days after birth (colostrum) is higher in proteins, vitamins A, B12, and K, and immunoglobulins and lower in fat content than mature milk [42,76,88]. Rapid changes occur in breast milk composition during the first week postpartum, and it can be considered mature approximately 15 days after birth [76]. The volume and composition of breast milk are not homogeneous; the milk differs over time and between species, and it also depends on the demands made by the infant and the nutritional status of the mother in the same species. Maternal intake influences the macro- and micronutrient content of breast milk, as well as its immunological properties [44,47,89]. Indeed, the amount of retinol concentration in milk seems to be independent of serum retinol levels. On the contrary, several reports have revealed a strong correlation between vitamin A and carotenoid content in the diet during pregnancy and lactation and the amount of these micronutrients secreted in breast milk [17,43,90,91]. Due to the positive relationship between dietary intake of carotenoids and breastmilk concentration, breastfeeding mothers should have a diet abundant in this micronutrient. Therefore, the recommended dietary allowance (RDA) is higher for pregnant and lactating women than for non-pregnant women [17,19,31,41,42,43,48,75,90,92].

The concentration of vitamin A in human milk decreases over the course of lactation; it is maximal in the colostrum and reaches a plateau in mature milk. In healthy mothers, the vitamin A concentration varies from 5 to 7 μM in colostrum, 3 to 5 μM in transitional milk, and 1.4 to 2.6 μM in mature milk. The higher concentrations of retinol in colostrum allow tissue stores in the newborn to be rapidly replenished after limited placental transfer during gestation [25,76,88,90,92]. The newborn has a small amount of retinol pools in the liver (range 0–0.34 μmol/g in normal birth weight babies), achieved through the placental route, with maternal milk as the most important source of vitamin A [37,44,45,74,75,76,77,78,79]. Preterm birth aggravates the situation, since vitamin A liver stores are even lower, as the placental route has been cut off. These babies show lower plasma levels of retinol and retinol binding protein (RBP) at birth compared with full-term newborns. To compensate this deficit in retinol liver pools, it was observed that transitional milk in prematurity had a significant increase in micronutrient levels [91]. Milk retinol concentration in colostrum is lower in mothers of preterm babies (~3 μM) than in mothers of full-term babies [25,91]; however, it increases in transitional milk to values similar to those observed in milk from mothers of full-term babies (3.5 μM). There are no differences in mature milk between preterm and full-term, with milk retinol concentration of ~2 μM in mothers of preterm babies [91]. In this context, it is noteworthy that retinol level in preterm milk seems to be independent of the degree of prematurity [91].

During the first 6 months of life, infants from well-nourished mothers who are exclusively breastfed will accumulate around 310 μmol of vitamin A in the liver, approximately 60-fold higher than vitamin A accumulated throughout gestation by the transplacental pathway [25,88,90]. In fact, it has been reported that partial breastfeeding continues to provide a significant proportion of the recommended intake in infants after 6 months: 42% from 6 to 12 months and 61% during the second year in cases where vitamin A levels are low in the diet after weaning [78].

## 7. Deficiency of Vitamin A and Carotenoids during Pregnancy and Lactation

Vitamin A is necessary to maintain epithelial tissues, vision, and immune function. Breastfeeding from vitamin A-depleted mothers predisposes infants to the consequences of deficiency. Furthermore, VAD cannot be compensated by postnatal supplementation. As previously mentioned, pregnant and breastfeeding women are considered to be at risk of VAD in developing countries, where vitamin A in the diet is provided mainly as precursor carotenoids with lower bioavailability and efficiency to be converted into retinol. In this sense, several recent studies have shown that low dietary intake correlates with lower concentrations of vitamin A in milk of nursing mothers in Brazil [93,94], African countries [95,96,97,98,99], and Southeast Asia [26]. Thus, vitamin A supplementation for lactating mothers in these areas is regarded as a practical public health strategy to support maternal breastfeeding of newborns, more so of premature babies [25,40,90,92,100,101].

Serum retinol concentration is used to assess vitamin A status, with values below a cutoff of 0.70 μmol/L representing VAD, and below 0.35 μmol/L representing severe VAD. For pregnancy or lactation, the cutoff value is higher, and serum retinol concentration below 1.05 μmol/L reflects low vitamin A status among pregnant and lactating women [16]. The WHO Global Database estimated that clinical VAD (with night blindness and Bitot’s spots) and biochemical VAD (with serum retinol concentration <0.70 μmol/L) affected 7 and 219 million preschool-aged children, respectively [16]. Regarding estimations in pregnant women, 19.8 million had low vitamin A status (serum retinol or breast milk concentration < 1.05 μmol/L), of whom 7.2 million were deficient in vitamin A (<0.70 μmol/L) and 6.2 million experienced gestational night blindness. These estimates found that nearly two-thirds of the women with night blindness lived in South and Southeast Asia [16,77,102].

However, it is important to point out that even in Western countries, where vitamin A- and β-carotene-rich foods are normally available, there are groups at risk for low vitamin A levels [16,45,77,103,104]. One-fifth of the population in developed countries does not get the full recommended intake, with plasma and liver concentrations of vitamin A lower than those accepted as normal [104]. This situation can be aggravated by the increasingly common tendency to reduce fat intake in the diet and to engage in uncontrolled weight loss diets. Therefore, in these countries, it has been considered as subclinical vitamin A deficiency, which has increased markedly worldwide in the last decades [16,32,33,40]. Although calorie and protein content are largely unaffected by a woman’s diet, there are some micronutrients in breast milk that will only be at adequate levels if there is sufficient dietary intake. Nutritional supplements should be given to these mothers to fulfill the requirements of micronutrients such as vitamin A in newborns [19,48,103]. It is well established that maternal VAD in pregnancy leads to placental dysfunction, fetal loss, congenital malformations, and preterm birth. Vitamin A-deficient mothers will produce breast milk that is low in the vitamin, consequently triggering VAD in the offspring. During lactation, if breast milk does not provide the neonate with appropriate vitamin A levels, the immune system might be affected, raising the risk for infectious diseases. Indeed, respiratory tract infections and complications during viral infections (e.g., measles) are increased in children with VAD [45]. Also, lung development could be compromised, increasing the risk for bronchopulmonary disease [45]. Indeed, recommended vitamin A during pregnancy, and especially during lactation, increases considerably, an almost 2-fold increase compared to non-pregnancy in terms of RDA [18,31,37,45,74,77,92,102]. A low neonatal store not only increases the risk of infectious diseases in the perinatal period, but also could have a negative effect on health later on in life [45,46,47]. The most specific clinical effect and one of the first manifestations of VAD is xerophthalmia with nyctalopia. If untreated, this can progress to night blindness; depressed immunity; squamous metaplasia of mucous epithelium in several organs; hyperkeratosis; disturbances in cell differentiation, organ development, growth, and reproduction; increased risk of infection; and mortality [16,32,33,38,43,46,74,102]. Similarly, VAD during pregnancy in experimental animals led to the formation of hypoplastic organs or alterations in fetal organs, such as eyes, brain, kidneys, and lungs, and even to the death and resorption of fetuses, depending on the severity of the deficiency [105,106,107].

## 8. Conclusions

Vitamin A plays a role in mammary gland metabolism during lactation. On the one hand, RA is essential for mammary gland development and, later on, in secretory epithelia to achieve adequate milk production. On the other hand, retinoids, through the RARα-dependent signaling pathway, have also been shown to regulate, at least in part, the weaning process, where epithelial cell death is coupled with tissue remodeling. Nevertheless, RA should not be considered exclusively as a signaling molecule controlling the development of mammary tissue, but also as a micronutrient essential for fetal and neonatal development. In this sense, the fetal liver stores only a small amount of vitamin A during gestation; therefore, the neonate depends on the external supply from the mother’s milk. An appropriate dietary intake of retinol and β-carotene during pregnancy and lactation together with the intertissue flux of different nutrients in the gland during lactation influence vitamin A concentration in breast milk. This is important in the long term, not only for maternal health, but also for the offspring, because vitamin A is involved in postnatal physiological functions.
